# Global mRNA profiling reveals the effect of boron as a crop protection tool against *Sclerotinia sclerotiorum*

**DOI:** 10.1093/aobpla/plae056

**Published:** 2024-09-26

**Authors:** Natalie L Cale, Philip L Walker, Sanjana Sankar, Sean M Robertson, Olivia Wilkins, Mark F Belmonte

**Affiliations:** Department of Biological Sciences, University of Manitoba, Winnipeg, Manitoba, R3T 2N2, Canada; Morden Research and Development Centre, Agriculture and Agri-Food Canada, Morden, Manitoba, R6M 1Y5, Canada; Department of Biological Sciences, University of Manitoba, Winnipeg, Manitoba, R3T 2N2, Canada; Department of Biological Sciences, University of Manitoba, Winnipeg, Manitoba, R3T 2N2, Canada; Department of Biological Sciences, University of Manitoba, Winnipeg, Manitoba, R3T 2N2, Canada; Department of Biological Sciences, University of Manitoba, Winnipeg, Manitoba, R3T 2N2, Canada

**Keywords:** Boron, *Brassica napus*, canola, plant immunity, RNA sequencing, *Sclerotinia sclerotiorum*

## Abstract

*Sclerotinia sclerotiorum*, the causal agent of white mould, is a necrotrophic fungal pathogen responsible for extensive crop loss. Current control options rely heavily on the application of chemical fungicides that are becoming less effective and may lead to the development of fungal resistance. In the current study, we used a foliar application of boron to protect *Brassica napus* (canola) from *S. sclerotiorum* infection using whole-plant infection assays. Application of boron to aerial surfaces of the canola plant reduced the number of *S. sclerotiorum*-forming lesions by 87 % compared to an untreated control. Dual RNA sequencing revealed the effect of boron on both the host plant and fungal pathogen during the infection process. Differential gene expression analysis and gene ontology term enrichment further revealed the mode of action of a foliar boron spray at the mRNA level. A single foliar application of boron primed the plant defence response through the induction of genes associated with systemic acquired resistance while an application of boron followed by *S. sclerotiorum* infection-induced genes associated with defence response-related cellular signalling cascades. Additionally, in *S. sclerotiorum* inoculated on boron-treated *B. napus*, we uncovered gene activity in response to salicylic acid breakdown, consistent with salicylic acid-dependent systemic acquired resistance induction within the host plant. Taken together, this study demonstrates that a foliar application of boron results in priming of the *B. napus* plant defence response, likely through systemic acquired resistance, thereby contributing to increased tolerance to *S. sclerotiorum* infection.

## Teaser

*Sclerotinia sclerotiorum* , the causative agent of white mould, is one of the top pathogens of *Brassica napus* , responsible for extensive losses in crop yield. In this study, we investigated the use of foliar-applied boron as a crop protection tool. RNA sequencing revealed that boron is capable of protecting *B. napus* likely through both direct antagonism with *S. sclerotiorum* , in addition to priming the plant defence response. Additional studies into the use of foliar-applied boron in the field may result in alternative tools for growers to help protect crop species from fungal pathogens.

## Introduction

*Brassica napus* (canola, oilseed rape) is an internationally cultivated oilseed crop of immense agricultural and economic importance (Neik *et al.* 2017). Internationally, Canada is the largest *B. napus* producer, with 55 % of its production being exported to other countries (Carré and Pouzet 2014; Tan *et al.* 2024). *Brassica napus* is susceptible to a wide range of pathogens of both bacterial and fungal origin, including *Sclerotinia sclerotiorum* (Neik *et al.* 2017). Known as the causative agent of white mould or Sclerotinia stem rot, *S. sclerotiorum* is a necrotrophic fungal pathogen known to infect >600 plant species (Liang and Rollins 2018). *Sclerotinia sclerotiorum* is known for its capacity to cause devastating yield losses in crop species around the world, and is regarded as one of the most economically damaging pathogens of *B. napus* (Bolton *et al.* 2006; Derbyshire and Denton-Giles 2016). Due to its complex lifecycle and capacity to produce sclerotia capable of overwintering in the soil, effective control of the fungus has proven difficult (Liang and Rollins 2018; Wytinck *et al.* 2022; Hossain *et al.* 2023). To date, no cultivar of *B. napus* has been identified to be truly resistant to Sclerotinia stem rot. While some partially resistant cultivars exist, improved resistance to *S. sclerotiorum* using traditional breeding strategies has proved (Bolton *et al.* 2006; Khot *et al.* 2011; Girard *et al.* 2017; Wang *et al.* 2019; Chen *et al.* 2023). The primary control method against *S. sclerotiorum* is the application of chemical fungicides; however, due to concerns associated with fungicide resistance and environmental/ecological sustainability, there exists an immediate need to develop new strategies to combat this fungal pathogen (Wu *et al.* 2013; Duke *et al.* 2017).

As *S. sclerotiorum* infects *B. napus*, the fungal pathogen uses senescing petals as a nutrient source where the growing hyphae then penetrate host tissues to colonize the vasculature, eventually leading to lodging (Derbyshire and Denton-Giles 2016; Girard *et al.* 2017; Wytinck *et al.* 2022). Due to the rapid attack and degradation of host tissues by *S. sclerotiorum*, the plant immune response is generally insufficient to defend against infection (Wytinck *et al.* 2022). Plants have evolved complex mechanisms to defend themselves upon pathogen attack. As such, the host plant response to pathogenic fungi relies on a variety of signalling processes and resistance/immunity pathways. For example, activation of the innate immune system occurs when the presence of the pathogen is recognized through extracellular pathogen-associated molecular patterns (PAMPs). *Sclerotinia sclerotiorum* is also detected via pattern recognition receptors (PRRs), specifically receptor protein kinases (RLKs), or through the detection of pathogen effectors via nucleotide-binding leucine-rich repeat (NLR) receptors (Song *et al.* 2021). Recognition of PAMPs via PRRs elicits pattern-triggered immunity (PTI), whereas recognition of pathogen effectors via NLRs elicits effector-triggered immunity (ETI). It is generally accepted that PTI confers a lower level of immunity effective against non-adapted pathogens, whereas ETI is more effective against host-adapted pathogens as its associated immunity is more robust (Pruitt *et al.* 2021). The two immune pathways are not found to be mutually exclusive, with co-induction having led to increased pathogen resistance, and PTI even having been found to be necessary for a successful immune response as a result of ETI (Pruitt *et al.* 2021; Walker *et al.* 2022). Following pathogen recognition, early defence responses are initiated such as signal transduction and mitogen-activated protein kinase (MAPK) cascades, which in turn lead to calcium ion import, reactive oxygen species (ROS) burst and regulation of phytohormone signalling pathways that allow for defence gene induction (Song *et al.* 2021; Walker *et al.* 2022; Wytinck *et al.* 2022).

Following a targeted immune response, plant systemic resistance may be obtained via two discrete pathways: (i) systemic acquired resistance (SAR) and (ii) induced systemic resistance (ISR) (Duke *et al.* 2017). When SAR is induced, areas distal from the infection site take on a heightened immune state, undergoing large-scale transcriptional changes that leave them primed for a more rapid and robust response following pathogen infection (Jung *et al.* 2009; Zeier 2021). SAR activation is often characterized by increased levels of the defence hormone, salicylic acid (SA), in addition to increased pathogenesis-related (PR) protein expression systemically (Pieterse *et al.* 2014; Bernsdorff *et al.* 2016). While increased systemic resistance to pathogen attack is also seen as a result of induced ISR, the two resistance pathways operate through distinct mechanisms where ISR is seen to be effective without the accumulation of PR proteins, and is mediated in an SA-independent signalling pathway (Pieterse *et al.* 2014). Additionally, ISR is commonly seen to be initiated through interactions with plant roots by non-pathogenic rhizobacteria or fungi, whereas SAR is commonly activated following pathogen attack or as a result of defence response-related molecules/hormones (Pieterse *et al.* 2014; Duke *et al.* 2017).

Boron is a metalloid and plant micronutrient essential for growth and development (García-Sánchez *et al.* 2020). The boron requirement of plants differs by species and cultivar, however, is considered high in *B. napus* compared to other crops (Zhang *et al.* 2014). Although known to impact various metabolic processes, the main function of boron in plants is the maintenance of cell wall structure through the formation of rhamnogalacturonan-II-B complexes that stabilize pectin networks (García-Sánchez *et al.* 2020). Recently, boron application has been found to mitigate symptoms of bacterial and fungal disease in crops including *B. napus* (Deora *et al.* 2011; Ni and Punja 2020), *Solanum tuberosum* (potato) (Frenkel *et al.* 2010), *Solanum lycopersicum* (tomato) (Frenkel *et al.* 2010; Jiang *et al.* 2016; Martinko *et al.* 2022), *Musa* spp. (banana) (Dong *et al.* 2016) and *Vitis vinifera* (grapevine) (Rolshausen and Gubler 2005). While the use of boron as a crop protection tool has been investigated in these previous studies, it remains to be understood how this interaction occurs at the level of the transcriptome.

In the current study, we investigate the effects of a foliar application of boron on *B. napus* in the presence and absence of *S. sclerotiorum* infection. Discriminating doses of foliar-applied boron revealed this micronutrient was capable of protecting *B. napus* against *S. sclerotiorum* infection at the whole-plant level. Complimentary *in vitro* assays showed that boron acts as a direct antagonist to *S. sclerotiorum* growth. RNA sequencing results revealed large-scale transcriptomic changes in *B. napus* following a foliar application of boron, including priming of the plant defence response, likely through signal transduction cascades, and SAR. Together, these results show that a foliar application of boron has the potential to protect *B. napus* against *S. sclerotiorum* and that this interaction occurs both through direct antagonism and through plant defence priming.

## Materials and Methods

### *In vitro* assays

Potato dextrose agar (BD Difco) was prepared as per manufacturer’s instructions and supplemented with the chemical fungicide boscalid (10 ppm) as a positive control, or boron at concentrations of 0.10, 0.25 and 0.50 % (16, 40 and 80 mM H_3_BO_3_) before being poured into Petri plates and allowed to solidify. The centres of the plates were then inoculated with either *S. sclerotiorum* or *B. cinerea.* As inoculant, 10 µL of a 1 × 10^6^ ascospores per mL in potato dextrose broth, and 10 µL of a 2.0 × 10^6^ ascospores per mL in 25 % glycerol solution were used for *S. sclerotiorum* and *Botrytis cinerea*, respectively. Petri plates were sealed and incubated at room temperature in dark conditions for 8 days to allow for fungal growth.

### *Brassica napus* growth conditions

Seeds of *B. napus* cv. Westar were sown in Sunshine growing mix #4 and grown for the duration of the experiment under long day (16-h light, 8-h dark) greenhouse conditions (Sungro, Agawan, MA, USA). At the trifoliate stage, plants were transplanted to pots sized 8.5 cm × 8.5 cm × 9 cm, with 1.5–2 g Osmocote® fertilizer added atop the soil (The Scotts Company LCC, Maryville, OH, USA). Plants were grown to the 30 % flowering stage prior to treatment and subsequent inoculation with *S. sclerotiorum*.

### Detached leaf infection assays

Ascospores of *S. sclerotiorum* were sourced and stored according to Walker *et al.* (2022). Ascospores were suspended in potato dextrose broth at a concentration of 1 × 10^6^ spores per mL and Silwet L-77 (0.02 % v/v) as a surfactant. Harvested senescing *B. napus* petals were arranged on a sterile Petri dish, and 10 µL of ascospore suspension was pipetted onto each petal before the dish was sealed with Parafilm. Petals were incubated at room temperature for 48 h to allow for ascospore germination before being used as inoculant.

Whole *B. napus* leaves of plants at the 30 % flowering stage were removed from the plant and placed in Petri dishes lined with wet paper towel to maintain relative humidity. Leaves were sprayed with 0.10, 0.25, 0.5 or 1.0 % boric acid (H_3_BO_3_), corresponding to 16, 40, 80 and 160 mM, respectively. Boric acid solutions, hereafter referred to as boron, were compared against distilled water as a negative control, and 10 ppm boscalid, a Group 7 succinate dehydrogenase (SDH) inhibitor fungicide, as a positive control. As a surfactant, Silwet L-77 (0.03 % w/v) was added to each treatment immediately prior to spraying. Treatments were allowed to dry on the leaves before the Petri dishes were sealed and incubated for 24 h at room temperature prior to inoculation. After 24 h, leaves were inoculated by placing one infected petal onto the midvein of the leaves. Petri plates were then re-sealed, and leaves were left to incubate for 48 h at room temperature to allow for infection to progress (Wytinck *et al.* 2022; Walker *et al.* 2023). At this time, leaves were imaged and lesion area was calculated using the software ImageJ. In the case of phytotoxicity assays, plants did not undergo inoculation, and simply remained in Petri dishes for 72 h before being imaged for visual symptom assessment. Jitter bar plots for these measures were made using GraphPad Prism version 9.5.1 (GraphPad Software, San Diego, CA, USA).

### Whole-plant spray inoculations

Based on the results of the detached leaf assays, two treatments were compared to an untreated control (UTC): 10 ppm boscalid and 0.25 % boron. Silwet L-77 (0.06 % v/v) was added as a surfactant to each treatment immediately prior to spraying. Treatments were randomly assigned to plants, with 4.5 mL of each treatment sprayed per plant. Once treated, plants were left to dry for 24 h.

### *Sclerotinia sclerotiorum* inoculation of *B. napus* whole plants

*Sclerotinia sclerotiorum* ascospore suspension was prepared in the same manner as described above. Plants were sprayed with inoculum 24 h following the treatment spray, with 4.0 mL of inoculum sprayed per plant. Both uninoculated and inoculated plants were then placed in a 4 × 6 × 4 ft humidity chamber, whose interior had been misted with tap water, for 4 days to achieve humidity levels of 90–100 %. Inoculated and uninoculated plants were separated within the chamber such that inoculated petals would not come into contact with uninoculated plants. At this time point, ~10 representative leaves per treatment were randomly selected from the plants for image capture for the determination of lesion area and the number of lesion-forming petals. This assay was repeated 10 times, with ~10 plants per treatment in each assay. Statistical significance was determined using a Kruskal–Wallis and *post hoc* Dunn’s test with *P*-values adjusted for false discovery rate (FDR) using the Benjamini–Hochberg method. Similar to the detached leaf infection assays, ImageJ and GraphPad Prism were used. At this same time point, tissue was taken for molecular analysis, with whole infected leaves being removed from the plant and immediately flash-frozen using liquid nitrogen.

### RNA isolation, library preparation and RNA sequencing

Leaves from at least three different plants were pooled together for a single biological replicate. A total of three biological replicates were used for RNA sequencing. Tissue was ground to a fine powder using liquid nitrogen and a mortar and pestle. RNA was isolated using Qiagen RNeasy Plant Minikit as per manufacturer’s instructions with the addition of an on-column DNAse step using Qiagen DNAse (Qiagen, Toronto, ON, Canada). cDNA libraries were constructed by Genome Québec following their mRNA stranded library preparation protocol. Paired-end 100-base pair reads were sequenced for a minimum of 25 million reads per library on the Illumina NovaSeq 6000 sequencing system at Genome Québec (Montréal, QC, Canada). All sequencing data are found at the Gene Expression Omnibus, under accession GSE264324.

### RNA-seq analysis

Raw read processing was carried out using computing clusters available through Compute Canada and the Digital Research Alliance of Canada (www.aliancecan.ca). Sequence read quality was assessed with FastQC prior to read alignment (Andrews 2010). Paired-end reads were aligned to the *B. napus* genome v5.0 (GenBank assembly accession: GCA_000751015.1; Chalhoub *et al.* 2014) and the *S. sclerotiorum* genome (RefSeq assembly accession: GCF_000146945.2; Amselem *et al.* 2011) using HISAT2 (Kim *et al.* 2019). Transcript abundance was determined using featureCounts (Liao *et al.* 2014). Filtering of low counts, normalization of reads, determination of differentially expressed genes (DEGs) and principle component analysis were done in R (R Core Team 2021) using DESeq2 (Love *et al.* 2014), ashr (Stephens 2017) and plotted using ggplot2 (Wickham 2011). Genes with counts lower than 100 across all samples were filtered prior to proceeding with normalization. Raw sequence read counts were normalized using the median of ratios method in DESeq2 (Anders and Huber 2010). DEGs were called with a *P*-value < 0.05 when adjusted for FDR by the Benjamini–Hochberg method (Benjamini and Hochberg 1995). Gene ontology (GO) term enrichment was carried out on gene sets using ShinyGO V0.77 (Ge *et al.* 2020). Gene ontology and DEG lists [see Supporting Information—Supplemental_Dataset_1], raw counts aligned to *B. napus* [see Supporting Information—Supplemental_Dataset_2] and raw counts aligned to *S. sclerotiorum* [see Supporting Information—Supplemental_Dataset_3] are provided as supplemental data sets.

### Fungal load qPCR

To determine fungal load in infected samples, genomic DNA was first extracted using a modified cetyltrimethylammonium bromide method (Porebski *et al.* 1997). We used the *S. sclerotiorum* 18S rDNA as the target for relative fungal load using qPCR as described in Wytinck *et al.* (2022). Primer sequences and target loci can be found in Supporting Information—Table S1. SsoFast EvaGreen Supermix was used as per manufacturer’s instructions (Bio-Rad Laboratories, Hercules, CA, USA).

## Results

### Boron acts as a direct antagonist to *S. sclerotiorum* and *B. cinerea*

We first conducted *in vitro* assays to better understand the effect of boron on the growth response of *S. sclerotiorum* or the close fungal relative, *B. cinerea* [see Supporting Information—Fig. S1]. Eight days post-inoculation, no *S. sclerotiorum* growth was observed at any boron concentration, compared to minimal growth on boscalid plates. Extensive fungal growth extending to the plate margin was observed on control plates. This same assay was run using *B. cinerea* spores. While a reduction in mycelial growth was observed when *B. cinerea* spores were exposed to 0.25 and 0.50 % boron, boscalid was the most effective at restricting mycelial growth.

### Foliar applications of boron reduce symptoms of disease in *B. napus* infected with *S. sclerotiorum*

Prior to conducting infection assays, foliar applications of boron were applied to *B. napus* leaves which were then monitored for symptoms of boron toxicity. Detached leaf toxicity assays revealed that boron foliar spray concentrations at and below 0.25 % failed to display symptoms of boron toxicity after 3 days. Application of 0.50 % boron resulted in toxicity symptom development, namely leaf chlorosis and marginal necrosis [see Supporting Information—Fig. S2]. No phytotoxicity symptoms were observed in the control or 10 ppm boscalid-treated leaves. In addition to toxicity assays, detached leaf infection assays were conducted to determine the concentration of boron required to reduce *S. sclerotiorum* lesion size. Both 0.25 % and 0.50 % boron foliar applications resulted in statistically significant reduction in lesion area compared to the control, with percent reductions in lesion area of 93.2 % and 94.5 %, respectively [see Supporting Information—Fig. S3]. Combining the results of the detached leaf phytotoxicity assay with those of the detached leaf infection assay, 0.25 % boron was chosen as the ideal treatment given its capacity to reduce lesion development in the absence of phytotoxicity.

**Figure 1.**
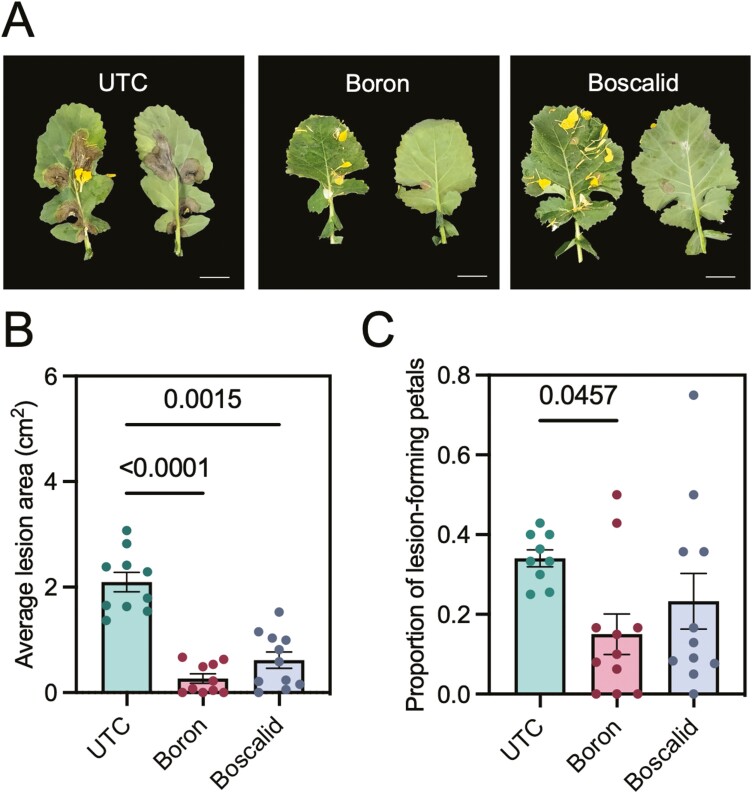
Response of *B. napus* (cv. Westar) to *S. sclerotiorum* following foliar applications of boron or boscalid. (A) Lesion development 4 days post-inoculation with *S. sclerotiorum* ascospores. Scale bar = 2 cm. Average lesion area (B) and proportion of lesion-forming petals (C) per leaf of UTC, boron- or boscalid-treated plants inoculated with *S. sclerotiorum*. Significance determined using a Kruskal–Wallis and *post hoc* Dunn’s test with *P*-values FDR adjusted using the Benjamini–Hochberg method. *n* = 8–10 infected representative leaves per treatment. Error bars correspond to standard error.

Next, we conducted whole-plant *B. napus* infection assays with *S. sclerotiorum* following treatment with boron or boscalid (Fig. 1A). At 4 days post-inoculation, both boron- and boscalid-treated plants showed a reduction in lesion area (Fig. 1B) as well as proportion of lesion-forming petals (Fig. 1C). For example, both foliar treatments significantly reduced lesion area, with boron and boscalid reducing lesion area by factors of 87 % and 71 %, respectively (Fig. 1B). The proportion of lesion-forming petals was also reduced by 56 % when plants were treated with boron, or 32 % when plants were treated with boscalid (Fig. 1C). Additionally, we measured *S. sclerotiorum* fungal load using 18S rDNA markers between the boron- or boscalid-treated plants and the UTC [see Supporting Information—Fig. S4]. Boron-treated tissues were found to have a 41 % reduction in average fungal load compared to the UTC while boscalid reduced fungal load by an average of 34 %.

### Global gene expression analysis reveals boron and boscalid elicit unique transcriptional responses in *B. napus*

**Figure 2.**
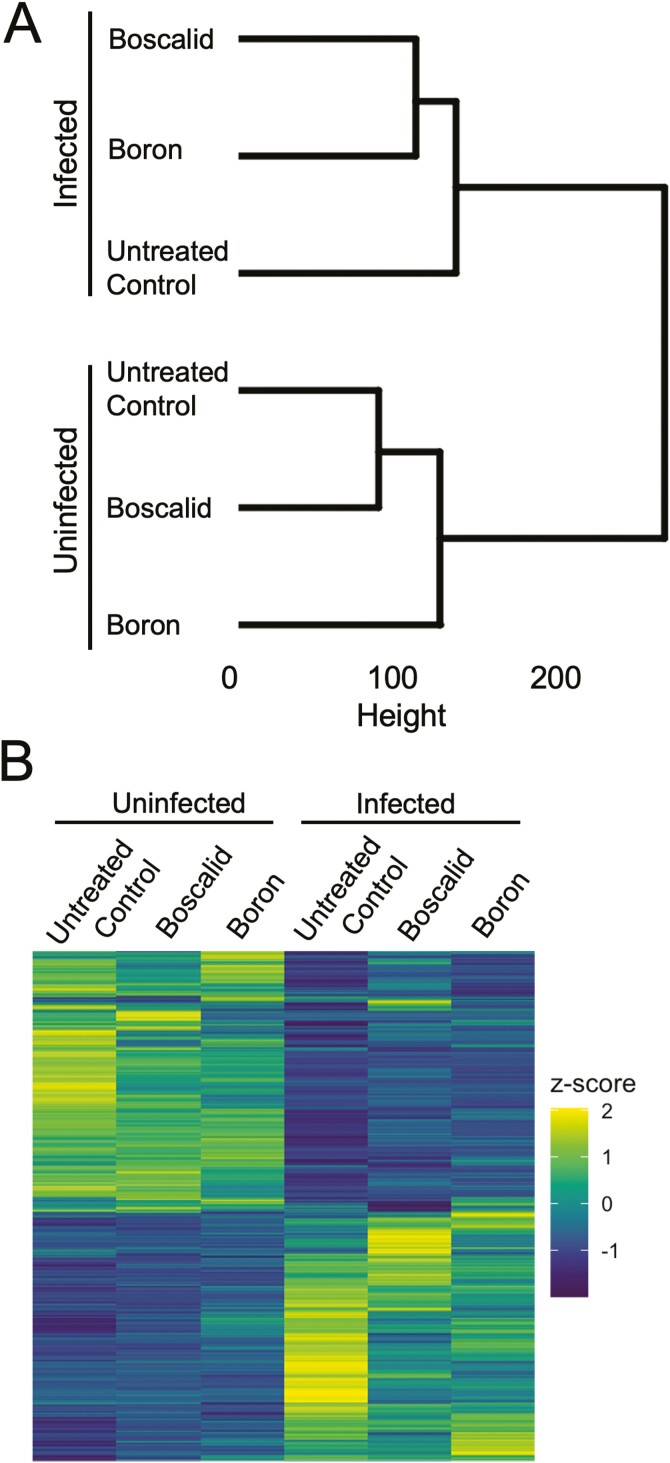
Global mRNA sequencing and differential gene expression (DEG) analysis. (A) Hierarchical clustering of the top 20 000 most variable genes of *B. napus* treated with boron or boscalid in the presence or absence of *S. sclerotiorum*. Branch height corresponds to distance between clusters (Euclidian distance). (B) Heat map visualizing the 33 357 genes found to be significantly differentially expressed (FDR < 0.05) in response to treatment and infection.

To better understand how *B. napus* treated with either boron or boscalid responded to *S. sclerotiorum*, we profiled global gene expression patterns in response to both foliar treatment and infection (Fig. 2). Hierarchical clustering of the top 20 000 most variably expressed genes between treatment and infection statuses revealed the largest division between samples was at the infection level (Fig. 2A; see Supporting Information—Fig. S5). Within the uninfected group, UTC tissues grouped more closely with boscalid-treated *B. napus* compared to plants treated with boron. Within the infected group, boron- and boscalid-treated tissues subjected to *S. sclerotiorum* infection grouped more closely together. Differential gene expression analysis in response to treatment and infection revealed 33 357 significantly DEGs with FDR-adjusted *P*-value < 0.05. Heat map of these DEGs revealed large shifts in gene activity in response to both treatment and infection (Fig. 2B).

### Differential gene expression analysis reveals foliar application of boron primes plant defence responses at the mRNA level

**Figure 3.**
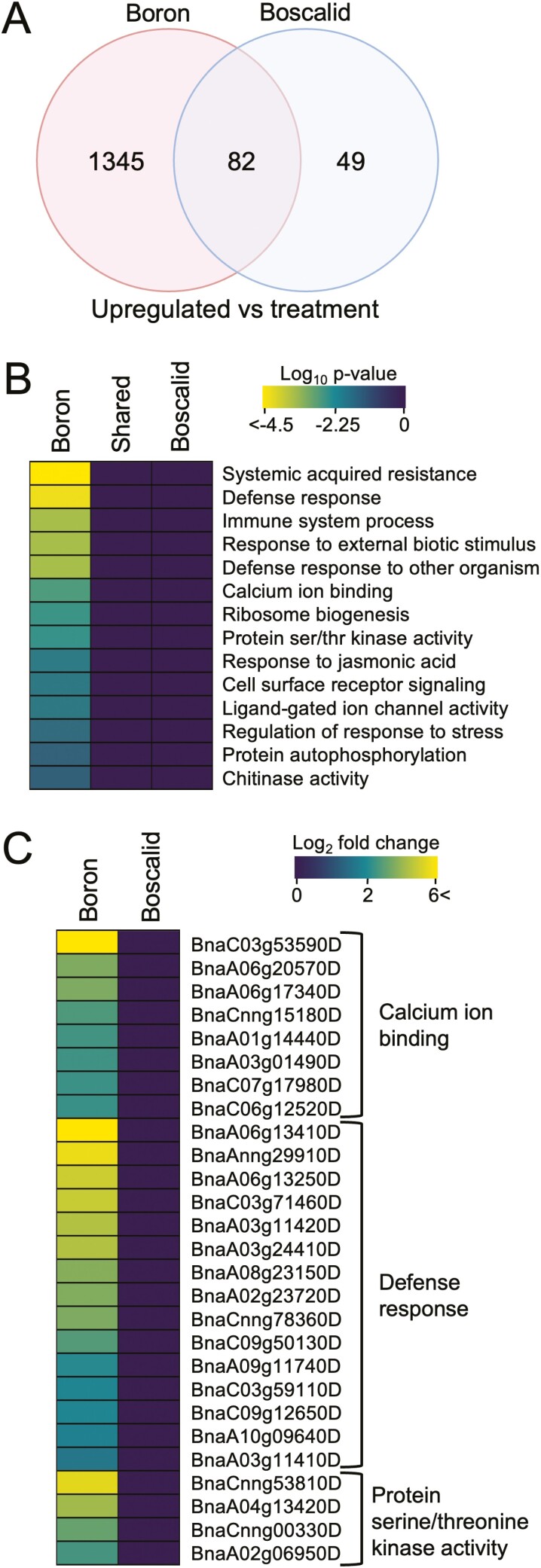
Differential gene expression and GO term enrichment of *B. napus* treated with boron or boscalid. (A) Venn diagram of significantly upregulated DEG sets (FDR < 0.05) in response to treatment with boron or boscalid compared to the untreated control. (B) Heat map of significantly enriched GO terms (FDR < 0.05) identified from the treatment-specific gene subsets. (C) Heat map of significantly DEGs (FDR < 0.05) belonging to enriched GO terms following boron treatment. Brighter yellow colour indicates a greater fold change.

We also studied the genes and biological processes that respond to foliar treatments in *B. napus* and conducted a differential expression and GO term enrichment analysis (Fig. 3). In response to foliar boron treatment, we identified 1345 specific significantly upregulated DEGs, whereas 49 genes were induced specifically in response to boscalid treatment (Fig. 3A). GO term enrichment revealed boron-treated leaves to be enriched in genes pertaining to biological processes associated with plant defence response priming (defence response *P* = 5.5 × 10^−5^ and immune process *P* = 0.00035), calcium ion binding (*P* = 0.0026), protein ser/thr kinase activity (*P* = 0.0045) and chitinase (*P* = 0.04); and plant defence hormones/pathways (SAR *P* = 2.6 × 10^−5^, jasmonic acid *P* = 0.013). None of the above-mentioned GO terms were enriched for in the boscalid- or control-treated *B. napus* (Fig. 3B).

We then compared gene activity of DEGs unique to foliar boron treatment and that encompassed these enriched GO terms (Fig. 3C). Boron treatment increased the accumulation of many *CALMODULIN-LIKE PROTEINS* (*CML*) transcripts, specifically *CML47*, *-48*, *-49*, *-50*, *-43* and -*46*, with fold changes in expression ranging from 5.3 (*CML46*) to 70.8 (*CML47*) times greater than the UTC. Additionally, genes involved in SAR and plant defence response had increased transcript abundance following treatment of boron. These genes included, *PATHOGENESIS-RELATED THAUMATIN SUPERFAMILY PROTEIN* (*PR5*) with a fold change of 83.0, *NPR1-LIKE PROTEIN 3* (*NPR3*) with a fold change of 12.25, *NIM1-INTERACTING PROTEIN 1* (*NIMIN1*) with a fold change of 11.5, *DEFECTIVE IN INDUCED RESISTANCE 1* (*DIR1*) with a fold changes of 3.7 and 3.3, and various *JASMONATE-ZIM DOMAIN* (JAZ) proteins that included *JAZ1*, *-5*, *-8* and *-10* with fold changes of 30.1, 21.9, 4.0 and 28.9, respectively.

We also studied DEGs downregulated in response to foliar treatment of boron or boscalid. We identified 751 downregulated DEGs that responded specifically to boron, while 88 DEGs were downregulated in response to boscalid, and 20 DEGs were shared between the two treatments [see Supporting Information—Fig. S6A]. GO enrichment of downregulated genes revealed processes associated with cell wall organization like pectin and polysaccharide catabolic process (*P* = 1.6 × 10^−5^ and *P* = 2.7 × 10^−5^, respectively), galacturonan metabolic process (*P* = 2.3 × 10^−5^), cell wall organization (*P* = 1.3 × 10^−4^) and signal transduction processes like protein serine-threonine kinase activity (*P* = 0.032) and calcium ion binding (*P* = 0.042). Shared to both treatments was the downregulation of plant cell wall biogenesis (*P* = 0.047). None of the above-mentioned GO terms were specifically downregulated in response to boscalid treatment [see Supporting Information—Fig. S6B].

### Differential gene expression analysis reveals boscalid foliar treatment leads to increased cellular signalling during infection by *S. sclerotiorum* at the mRNA level

**Figure 4.**
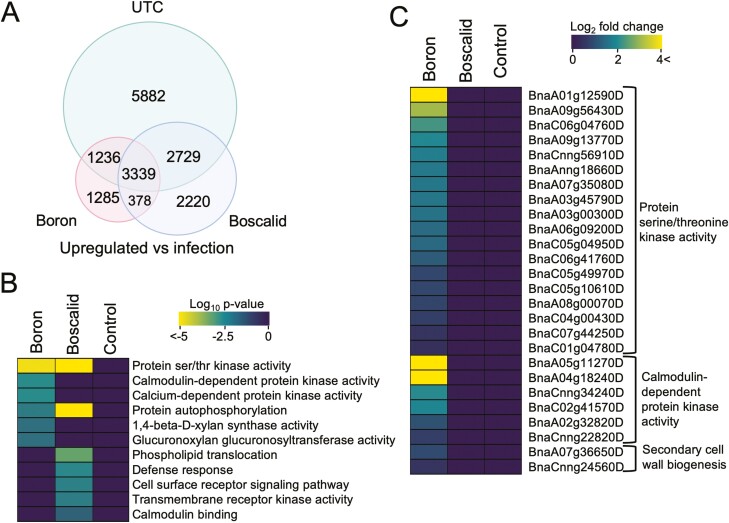
Differential gene expression and GO term enrichment of *B. napus* treated with boron or boscalid and in response to *S. sclerotiorum* infection. (A) Venn diagram of significantly upregulated DEG sets (FDR < 0.05) of infected tissue treated with boron or boscalid compared to the untreated control. Circle sizes are scaled to reflect the number of genes within them. (B) Heat map of a subset of significantly enriched GO terms (FDR < 0.05) identified from the treatment-specific gene list. (C) Heat map of significantly DEGs (FDR < 0.05) belonging to enriched GO terms following boron treatment and during infection.

Differential gene expression analysis identified gene sets that were upregulated in response to both foliar treatment and infection with *S. sclerotiorum* (Fig. 4). We uncovered 5882 upregulated DEGs specifically expressed in control tissues compared to 2220 DEGs that respond specially to boscalid treatment, and 1285 DEGs that respond specifically to foliar application of boron (Fig. 4A). Enriched GO terms specific to boscalid foliar application were heavily involved with signal transduction and early pathogen detection like protein ser/thr kinase activity (*P* = 1.3 × 10^−29^), protein autophosphorylation (*P* = 8.0 × 10^−21^), defence response (*P* = 0.0042) and cell surface receptor signalling (*P* = 0.0061). Plants treated with boron were enriched for GO terms associated with calcium-dependent signalling processes like calcium-dependent protein ser/thr kinase activity (*P* = 0.0036), calmodulin-dependent protein kinase activity (*P* = 0.0036) and cell wall maintenance like 1,4-beta-D-xylan synthase activity (*P* = 0.014) and glucuronoxylan glucuronosyltransferase activity (*P* = 0.014). None of these GO terms were enriched in control plants (Fig. 4B).

We then compared transcript abundance of DEGs associated with enriched GO terms in response to boron treatment and subsequent infection (Fig. 4C). Kinases including *CYSTEINE RICH RLK-11* (*CRK11*), *PR5-LIKE RECEPTOR KINASE* (*PR5K*)*, CYSTEINE RICH RLK-5* (*CRK5*) and *MITOGEN-ACTIVATED PROTEIN KINASE KINASE KINASE 13* (*MAPKKK13*) were upregulated with expression fold changes of 4.6 × 10^6^, 4.9, 3.3 and 2.8, respectively. Additionally, calmodulin- and calcium-dependent kinases were seen to have increased transcript abundance profiles including *CALCIUM-DEPENDENT PROTEIN KINASE 24* and *-26* (*CPK24* and *CPK26*) and *CALMODULIN-LIKE DOMAIN PROTEIN KINASE 9* (*CDPK9*) with fold changes of 3.9 × 10^9^, 4.2 and 4.0, respectively.

We were next interested in understanding the genes and biological processes downregulated in response to both foliar treatment and *S. sclerotiorum* infection [see Supporting Information—Fig. S7]. In the UTC plants, we identified 4905 specific downregulated DEGs, whereas 1896 and 815 DEGs were specific to boscalid and boron treatment, respectively [see Supporting Information—Fig. S7A]. GO enrichment on these gene sets identified biological processes associated with protein degradation and calcium ion binding in plants treated with boron. In response to boscalid we identified GO terms associated with peroxisome-mediated protein breakdown, mitochondrial breakdown and autophagy of mitochondrion. None of the above-mentioned GO terms were specifically downregulated in the UTC [see Supporting Information—Fig. S7B].

### Differential gene activity of *S. sclerotiorum* in response to foliar treatment of boscalid or boron

**Figure 5.**
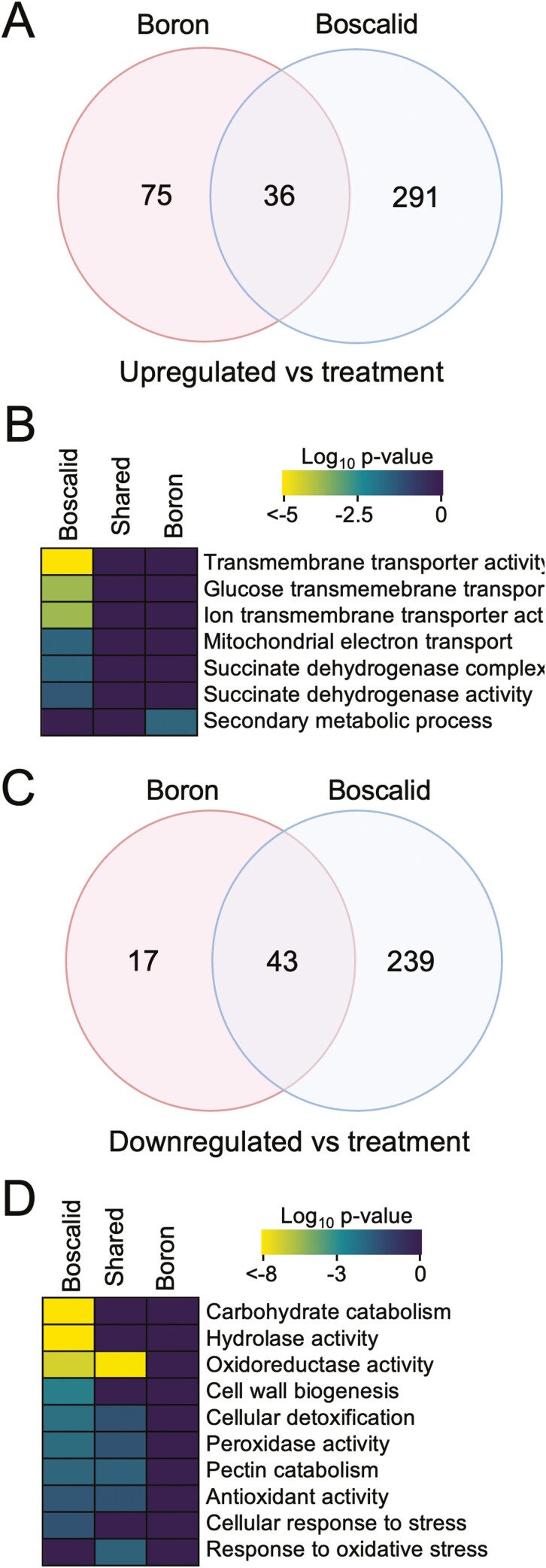
Differential gene expression and GO term analysis of *S. sclerotiorum* infecting boron- or boscalid-treated *B. napus*. (A) Venn diagram of significantly upregulated DEG sets (FDR < 0.05) of *S. sclerotiorum* on *B. napus* treated with boron or boscalid and compared to the untreated control. (B) Heat map of significantly enriched GO terms (FDR < 0.05) identified from the treatment-specific upregulated gene subsets. (C) Venn diagram of significantly downregulated DEG sets (FDR < 0.05) of *S. sclerotiorum* on *B. napus* treated with boron or boscalid and compared to the untreated control. (D) Heat map of significantly enriched GO terms (FDR < 0.05) identified from the treatment-specific downregulated gene subsets.

Differential expression analysis was then performed on *S. sclerotiorum* infecting boscalid- or boron-treated *B. napus.* Data analysis identified significantly up- and downregulated gene sets relative to *S. sclerotiorum* inoculated on untreated *B. napus* (Fig. 5). Here, 291 and 75 genes were significantly upregulated as a result of boscalid and boron treatments, respectively, with 36 upregulated genes shared across both treatments (Fig. 5A). GO term enrichment revealed increased transmembrane transport (*P* = 5.85 × 10^−6^) and SDH activity (*P* = 4.21 × 10^−2^) in *S. sclerotiorum* inoculated on boscalid-treated plants. *Sclerotinia sclerotiorum* inoculated on boron-treated plants revealed enrichment of secondary metabolic processes, more specifically, SA degradation (*P* = 2.19 × 10^−2^) (Fig. 5B). We also studied significantly downregulated genes where 239 and 17 downregulated genes were specific to boscalid and boron treatments, respectively, while 43 genes were downregulated in both treatments (Fig. 5C). GO enrichment of the shared gene set revealed enrichment of the ROS response, with enriched terms including oxidoreductase activity (*P* = 1.27 × 10^−8^), antioxidant activity (*P* = 2.79 × 10^−2^) and response to oxidative stress (*p* = 1.27 × 10^−2^) (Fig. 5D). Further, boscalid-specific genes showed similar terms involved in the ROS response while also showing enrichment of carbohydrate catabolism (*P* = 3.72 × 10^−12^) and cell wall biogenesis and organization (*P* = 2.28 × 10^−3^).

## Discussion

In the present study, we show that a foliar application of boron prior to fungal inoculation is capable of limiting symptoms of *S. sclerotiorum* infection in *B. napus*. *In vitro* and *in planta* studies combined with global mRNA profiling of gene activity underpinning this interaction in both the host plant and fungal pathogen revealed boron protects *B. napus* from *S. sclerotiorum* infection through both direct antagonism and the priming of host defence responses. Despite being an essential micronutrient for plant growth and development, boron present in excess is known to be phytotoxic causing chlorosis and necrosis and may result in reduced yields (Tombuloglu *et al.* 2015). While this study focused on the foliar application of boron, additional research is required prior to the use of boron for the purpose of crop protection at a field scale. Numerous site-specific variables must be considered prior to its commercial application such as existing soil and plant nutrition, soil/environment, pathogen threat, crop genetics, among others (Ryan 2005). Here, detached leaf infection assays were used to optimize boron application rates on *B. napus* to limit symptoms of phytotoxicity.

Protection of *B. napus* through application of boron was previously reported by Ni and Punja (2020), who applied boron-containing foliar fertilizers to *B. napus* leaves and observed a reduction in *S. sclerotiorum* lesion size. In addition to the *B. napus–S. sclerotiorum* pathosystem, boron has been found to protect numerous other crop species from both bacterial and fungal pathogens including the protection of *S. tuberosum* (potato) and *S. lycopersicum* (tomato) against *Phytophthora infestans* (Frenkel *et al.* 2010), *S. lycopersicum* against *Ralstonia solanacearum* and *Alternaria alternata* (Jiang *et al.* 2016; Martinko *et al.* 2022), *B. napus* against *Plasmodiophora brassicae* (Deora *et al.* 2011), *Musa* spp. (banana) against *Fusarium oxysporum* (Dong *et al.* 2016) and *V. vinifera* (grapevine) against *Eutypa lata* (Rolshausen and Gubler 2005). Although the effects of boron have been studied in variety of pathosystems, explorations into the underlying molecular mechanisms of protection remain unresolved. Frenkel *et al.* (2010) reported that SAR was induced as a result of boron application due to the pathogen resistance exhibited distally, although the authors did not perform any direct validation of SAR induction. Further, Jiang *et al.* (2016) found boron-treated *S. lycopersicum* infected with *R. solanacearum* to have elevated levels of ROS species, despite no molecular mechanisms being reported. Despite previous work describing the use of boron as a crop protection tool, none of the above research described this interaction at the mRNA level.

Pathogen detection brings together the concepts of PTI and ETI, where recognition of PAMPs or pathogenic elicitors can initiate unique molecular patterns (Pruitt *et al.* 2021; Dodds *et al.* 2024). In response to foliar boron application, we uncovered upregulation of genes whose products are known to be involved in pathogen perception and early defence response such as ser/thr RLKs and NLRs, CPKs and MAPKs (Dodds *et al.* 2024). While regarded as a necrotrophic pathogen, more recently the mode of *S. sclerotiorum* pathogenicity has been debated (Pruitt *et al.* 2021; Dodds *et al.* 2024). Current studies have challenged the classical view of the *S. sclerotiorum* necrotrophic lifestyle in reporting the possibility of a brief biotrophic phase occurring in early infection when *S. sclerotiorum* suppresses host SA-mediated SAR (Kabbage *et al.* 2015; Seifbarghi *et al.* 2017; Walker *et al.* 2023). In response to foliar boron treatment, we uncovered induction of SAR and upregulation of genes known to be implicated in SAR such as *DIR1* (*BnaA10g09640D* and *BnaA03g11410D*), *PR5* (*BnaA06g13410D*), *NIMIN1* (*BnaCnng78360D*) and *NPR3* (*BnaA02g23720D*). Previously, DIR1 has been characterized to be involved in long-distance SAR signalling to distal tissues, and has been reported in SAR-related defence priming of *B. napus*, leading to increased defence against *S. sclerotiorum* (Champigny *et al.* 2013; Duke *et al.* 2017). *PR5* was heavily upregulated in leaves treated with boron compared to the UTC. PR5, along with PR1 and PR2, are markers for SAR activation in *Arabidopsis thaliana* (Mishina and Zeier 2006; Gruner *et al.* 2013). In addition to the above SAR markers, SAR regulators NIMIN1 and NPR3 were seen to be induced in response to boron treatment. NIMIN1 and NPR3 are SA-responsive and work to fine-tune SAR, more specifically, in transcriptionally repressing defence genes, including SAR-associated PR proteins (Backer *et al.* 2019; Ding and Ding 2020; Hönig *et al.* 2023). While we observed SAR activation and at 5 days post-treatment, collection of plant material treated with boron at earlier time points should provide increased resolution of defence priming.

JAZ proteins JAZ1, -5, -8 and -10 were also identified as being upregulated in response to boron treatment. JAZ proteins play an essential role in the repression of MYC transcription factors involved in the expression of jasmonates (JA; Guo *et al.* 2018). Jasmonates are essential signalling molecules that contribute to biotic and abiotic stresses, coordinate developmental processes and work to balance the trade-off between growth and immunity (Chini *et al.* 2016; Guo *et al.* 2018; Howe *et al.* 2018). Previous studies have shown redundancy among the 13 known members of the *JAZ* family found in *A. thaliana*, with minimal phenotypic differences observable as a result of single *jaz* mutants (Chini *et al.* 2016; Guo *et al.* 2018). When cellular JA levels are low, JAZ proteins act as transcriptional repressors; however, when JA levels are elevated, JAZ proteins are degraded to allow for transcription of JA-responsive genes (Howe 2010; Kazan and Manners 2012). With the extensive crosstalk and known antagonism between SA and JA signalling, it remains possible that *JAZ* upregulation, and thus maintenance of low cellular levels of JA, is occurring as a result of SA-dependent SAR defence priming following foliar boron application (An and Mou 2011).

Several ser/thr and calmodulin-dependent RLKs were induced in response to both boron treatment and *S. sclerotiorum*. For example, the ser/thr RLK, *PR5K*, was significantly induced in our data set and has not only been found to be involved in plant non-self-recognition, but has also been reported to greatly impact the plant defence response against necrotrophic fungal pathogens (Sanabria *et al.* 2010; Walker *et al.* 2022). Previously, *A. thaliana* TDNA *pr5k* loss-of-function mutants demonstrated increased susceptibility to both *S. sclerotiorum* and *B. cinerea*, increasing fungal load by 310 % and 145 %, respectively (Walker *et al.* 2022). As a PRR, PR5K is capable of recognizing β-1,3-D-glucan, a fungal cell wall polysaccharide and PAMP, resulting in an immune response and SAR induction (Kombrink and Schmelzer 2001; Oliveira-Garcia and Deising 2013; Walker *et al.* 2022). Most heavily induced of the RLKs in response to boron treatment and infection was *CRK11*. CRK11 is a transmembrane ser/thr RLK whose expression is rapidly induced by SA and ROS (Czernic *et al.* 1999). It has been previously shown that ROS production is essential for the development of SA-dependent SAR (El-Shetehy *et al.* 2015). Taken together, foliar boron application elicits transcriptional upregulation of genes involved in pathogen perception and SAR induction and regulation which, when coupled with *S. sclerotiorum* infection, leads to upregulation of RLKs known to be involved in SAR such as *PR5K* and *CRK1*.

Boscalid is a broad-spectrum SDH inhibitor fungicide regulated to control fungi like *S. sclerotiorum* (Duarte Hospital *et al.* 2023). SDH inhibitors inhibit fungal respiration by binding the ubiquinone binding site of SDH involved in fungal mitochondrial electron transfer, thereby blocking electron transfer from succinate to ubiquinone (Fernández-Ortuño *et al.* 2017; Duarte Hospital *et al.* 2023). When infecting boscalid-treated *B. napus*, we uncovered increased SDH activity in *S. sclerotiorum* at the transcript level and is likely a response to the competition for the ubiquinone binding site of SDH resulting from boscalid application (Zhu *et al.* 2014; Duarte Hospital *et al.* 2023). Genes like *SS1G_04726* (2,3-dihydroxybenzoate decarboxylase) and *SS1G_08557* (salicylate hydroxylase), both known to be involved in the degradation of SA to catechol (Lubbers *et al.* 2021), were upregulated in *S. sclerotiorum* inoculated on *B. napus* treated with boron. Various fungal species, including *S. sclerotiorum*, have demonstrated the capacity to degrade SA produced by the host plant in an attempt to reduce SA-dependant plant defence mechanisms (Penn and Daniel 2013; Rocheleau *et al.* 2019). This finding, in addition to the upregulation of SAR markers, suggests that *B. napus* SA-dependent SAR is activated as a result of foliar boron application.

Taken together, we describe the protection of *B. napus* from *S. sclerotiorum* using a foliar application of boron. Our data show that treatment of *B. napus* with a foliar boron application leads to the priming of the plant defence response at the mRNA level, likely through SAR, providing increased tolerance to *S. sclerotiorum.* However, it remains to be understood how this interaction occurs over time, a question that can be answered through sampling at multiple time points. Additionally, it is still unclear how the plant may respond to foliar boron application and exposure to *S. sclerotiorum* in the long term or under field conditions. Additional studies exploring optimal concentrations of a boron-applied product in the field in addition to potential off-target effects are needed. Addressing these questions should lead to the development of new measures to protect crops in addition to directing future research regarding gene targets for crop improvement studies.

## Supporting Information

The following additional information is available in the online version of this article—

**Figure S1.** *In vitro* fungal activity of *S. sclerotiorum* and *B. cinerea* treated with boron and boscalid. Representative plates of *S. sclerotiorum* (top) and *B. cinerea* (bottom) growth for 8 days on potato dextrose agar plates amended with 0.10, 0.25, and 0.50 % boron and compared to an untreated negative control (left) and 10 ppm boscalid as a positive control (right).

Figure S1 alt text. Growth of *S. sclerotiorum* and *B. cinerea* on Petri plates containing growth media supplemented with various boscalid and boron at various concentrations.

**Figure S2.** Symptoms of phytotoxicity in *B. napus* detached leaves treated with sprayed water (untreated control), with increasing concentrations of boron, or with 0.10, 0.25, and 0.50 % boron and compared to an untreated negative control (left) and 10 ppm boscalid as a positive control (right). Leaves were treated 72 h prior to image capture. Scale bar = 5 cm.

Figure S2 alt text. Leaves of *B. napus* after being sprayed with boscalid and various concentrations of boron. All leaves appear green with the exception of the highest sprayed boron concentration of 0.50 %, which is displaying symptoms of chlorosis.

**Figure S3.** Average lesion area of control, boron- or boscalid-treated detached *B. napus* leaves inoculated with *S. sclerotiorum*. Significance determined using a Kruskal–Wallis and *post hoc* Dunn’s test with *P*-values false discovery rate adjusted using the Benjamini–Hochberg method. *P < 0.05, **P < 0.01, ***P < 0.001, ****P < 0.0001. *n* = 8–10. Error bars correspond to standard error.

Figure S3 alt text. Jitter bar graph showing *S*. *sclerotiorum* lesion area on *B. napus* leaves after foliar applications boscalid and various concentrations of boron compared to an untreated control. Bar graph also has significance markers present above bars.

**Figure S4.** Relative fungal load of *B. napus* treated with either boron or boscalid and then inoculated with *S. sclerotiorum*. Fungal load was determined measuring relative 18S rDNA abundance by qPCR. UTC, untreated control.

Figure S4 alt text. Bar graph showing relative abundance of *Sclerotinia* 18S rDNA between boron- or boscalid-treated *B. napus* compared to an untreated control.

**Figure S5.** Principal component analysis of *B. napus* (cv. Westar) plants following foliar applications of 0.25 % boron or 10 ppm boscalid and compared to a control treatment (untreated control) in the presence or absence of *S. sclerotiorum* infection considering the top 20 000 most variable genes.

Figure S5 alt text. Principle component analysis chart showing variance between individual replicates of samples considering treatment and infection status.

**Figure S6.** Differential gene expression and gene ontology (GO) term enrichment of *B. napus* treated with boron or boscalid. (A) Venn diagram of downregulated significantly differentially expressed gene sets (false discovery rate [FDR] < 0.05) in leaves treated with boron or boscalid compared to the untreated control. (B) Heat map of significantly enriched GO terms (FDR < 0.05) identified from the treatment-specific gene subsets.

Figure S6 alt text. Venn diagram and heat map showing the number of *B. napus* downregulated genes, and biological and molecular processes that respond to treatment with boron or boscalid.

**Figure S7.** Differential gene expression and gene ontology (GO) term enrichment of *B. napus* treated with boron or boscalid and in response to *S. sclerotiorum* infection. (A) Venn diagram of significantly downregulated differentially expressed gene sets (false discovery rate [FDR] < 0.05) in infected leaves treated with boron or boscalid compared to the untreated control. Circle sizes have been scaled to reflect the number of genes within them. (B) Heat map of significantly enriched GO terms (FDR < 0.05) identified from the treatment-specific gene subsets.

**Supporting_Information_Table_S1.docx:** *Sclerotinia sclerotiorum* fungal load qPCR primer sequence and associated efficiency.

**Supplemental_Dataset_1.xlsb:** Differential gene expression results, treatment-specific differential gene expression results and gene ontology term enrichment results for *B. napus* and *S. sclerotiorum*.

**Supplemental_Dataset_2.txt:** *Brassica napus* raw counts.

**Supplemental_Dataset_3.txt:** *Sclerotinia sclerotiorum* raw counts.

**Supporting_Information_Figures.pdf:** Supporting Information—Figs S1–S7.

plae056_suppl_Supplementary_Dataset_S1

plae056_suppl_Supplementary_Dataset_S2

plae056_suppl_Supplementary_Dataset_S3

plae056_suppl_Supplementary_Figures_S1-S7

plae056_suppl_Supplementary_Table_S1

## Data Availability

All RNA sequencing data are publicly available online via the Gene Expression Omnibus at the accession GSE264324.
