# Supplementary material for: Global mRNA profiling reveals the effect of boron as a crop protection tool against Sclerotinia sclerotiorum
Source: AoB Plants. 2024 Sep 26;16(6):plae056. doi: 10.1093/aobpla/plae056 (PMC11551614; doi:10.1093/aobpla/plae056)
Supplement: plae056_suppl_Supplementary_Figures_S1-S7 [file plae056_suppl_supplementary_figures_s1-s7.pdf]

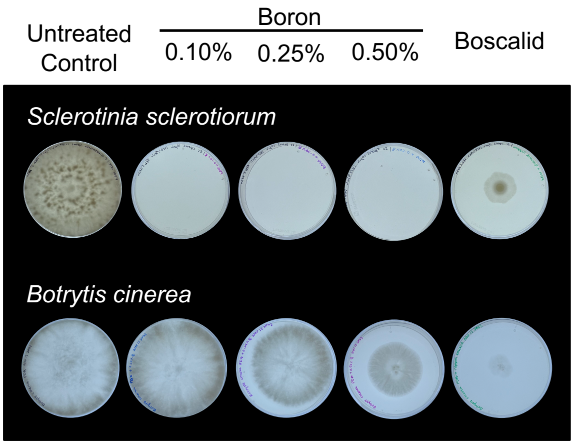

Figure S1. *In-vitro* fungal activity of *S. sclerotiorum* and *Botrytis cinerea* treated with boron and boscalid. Representative plates of *S. sclerotiorum* (top) and *B. cinerea* (bottom) growth for eight days on PDA plates amended with 0.10%, 0.25%, and 0.50% boron and compared to an untreated negative control (left) and 10 ppm boscalid as a positive control (right).

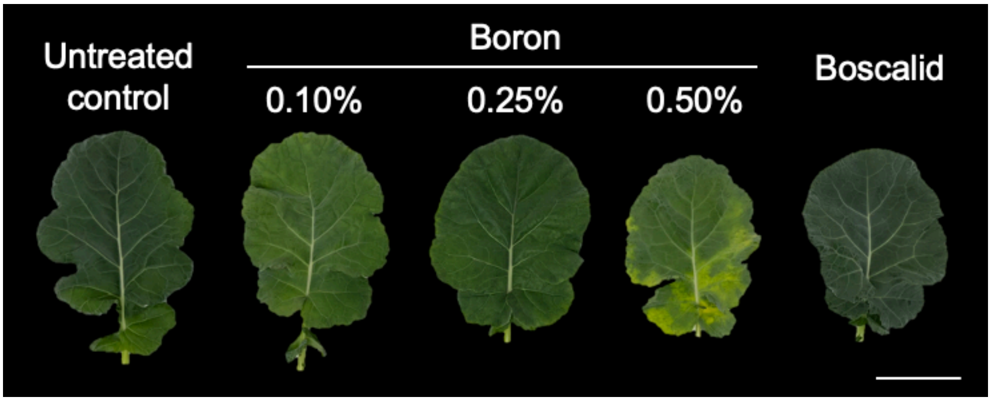

Figure S2. Symptoms of phytotoxicity in *B. napus* detached leaves treated with sprayed water (untreated control), with increasing concentrations of boron, or with 0.10%, 0.25%, and 0.50% boron and compared to an untreated negative control (left) and 10 ppm boscalid as a positive control (right). Leaves were treated 72 hours prior to image capture. Scale bar = 5cm.

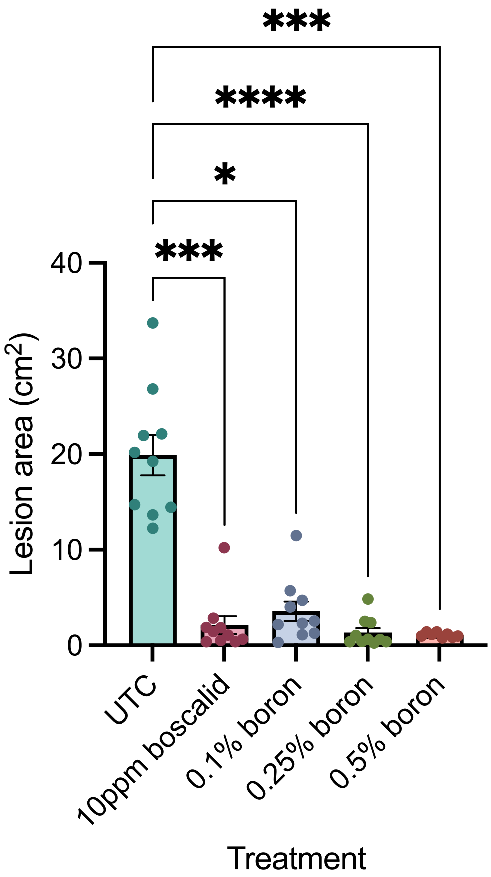

Figure S3. Average lesion area of control, boron, or boscalid treated detached *B. napus* leaves inoculated with *S. sclerotiorum*. Significance determined using a Kruskal-Wallis and post-hoc Dunn's test with p-values FDR adjusted using the Benjamini-Hochberg method. * = $p<0.05$, ** = $p<0.01$, *** = $p<0.001$, **** = $p<0.0001$. n = 8-10. Error bars correspond to standard error.

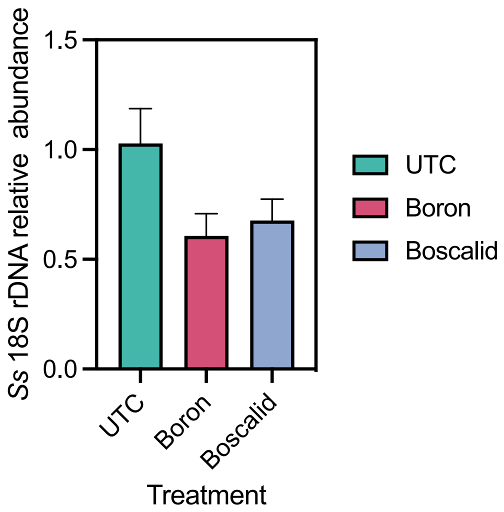

Figure S4. Relative fungal load of *B. napus* treated with either boron or boscalid and then inoculated with *S. sclerotiorum*. Fungal load was determined measuring relative 18S rDNA abundance by qPCR. Differences were not found to be statistically significant. UTC, untreated control.

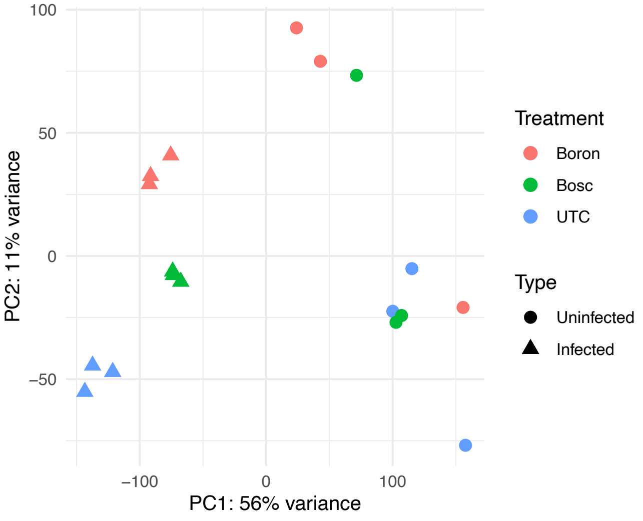

Figure S5. Principal component analysis of *B. napus* (cv. Westar) plants following foliar applications of 0.25% boron or 10m ppm boscalid and compared to a control treatment (UTC) in the presence or absence of *S. sclerotiorum* infection considering the top 20,000 most variable genes.

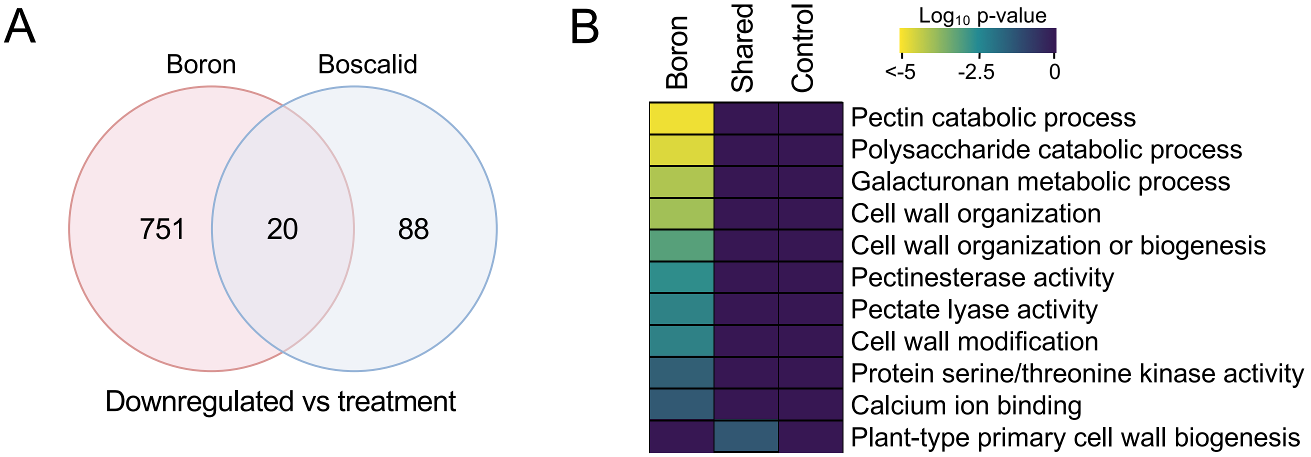

Figure S6. Differential gene expression and gene ontology term enrichment of *B. napus* treated with boron or boscalid. (A) Venn diagram of downregulated significantly differentially expressed gene sets (FDR<0.05) in leaves treated with boron or boscalid compared to the untreated control. (B) Heatmap of significantly enriched GO terms (FDR<0.05) identified from the treatment-specific gene subsets. A brighter yellow colour indicates greater statistical significance.

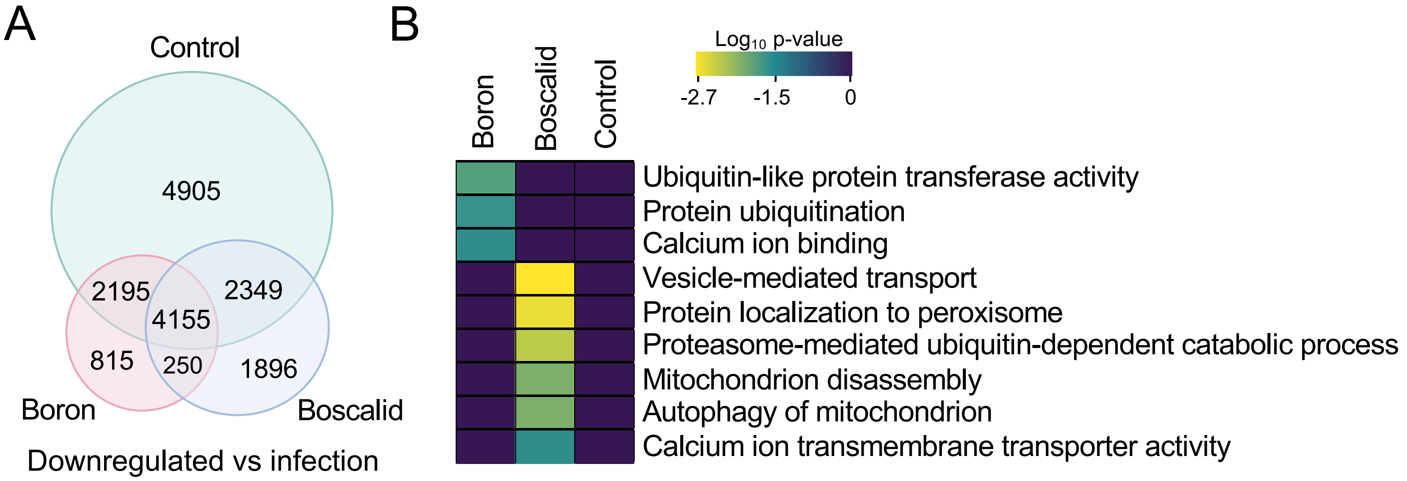

Figure S7. Differential gene expression and gene ontology term enrichment of *B. napus* treated with boron or boscalid and in response to *S. sclerotiorum* infection. (A) Venn diagram of significantly downregulated differentially expressed gene sets (FDR<0.05) in infected leaves treated with boron or boscalid compared to the untreated control. Circle sizes have been scaled to reflect the number of genes within them. (B) Heatmap of significantly enriched GO terms (FDR<0.05) identified from the treatment-specific gene subsets. A brighter yellow colour indicates greater statistical significance.
