# Supplementary material for: Global mRNA profiling reveals the effect of boron as a crop protection tool against Sclerotinia sclerotiorum
Source: AoB Plants. 2024 Sep 26;16(6):plae056. doi: 10.1093/aobpla/plae056 (PMC11551614; doi:10.1093/aobpla/plae056)
Supplement: plae056_suppl_Supplementary_Table_S1 [file plae056_suppl_supplementary_table_s1.docx]

Table S1. *S. sclerotiorum* fungal load qPCR primer sequence and associated efficiency.

|  | Gene name | Primer sequences (5’-3’) | Efficiency (%) |
| --- | --- | --- | --- |
| Ss 18S rDNA | *S. sclerotiorum*  *18S RIBOSOMAL rDNA* | F: AGCCGATGGAAGTTTG AGGC  R: CTCGTTGGCTCTGTCAG TGT | 104.84 |
